# Ultrafast Detection of Arsenic Using Carbon-Fiber Microelectrodes and Fast-Scan Cyclic Voltammetry

**DOI:** 10.3390/mi15060733

**Published:** 2024-05-31

**Authors:** Noel Manring, Miriam Strini, Gene Koifman, Jonathan Xavier, Jessica L. Smeltz, Pavithra Pathirathna

**Affiliations:** Department of Chemistry and Chemical Engineering, Florida Institute of Technology, 150 W. University Blvd, Melbourne, FL 32901, USA; nmanring2020@my.fit.edu (N.M.); mstrini2021@my.fit.edu (M.S.); gkoifman2021@my.fit.edu (G.K.); jxavier2020@my.fit.edu (J.X.); jsmeltz@fit.edu (J.L.S.)

**Keywords:** arsenic, carbon fiber microelectrodes, fast-scan cyclic voltammetry, real-time analysis, co-detection

## Abstract

Arsenic contamination poses a significant public health risk worldwide, with chronic exposure leading to various health issues. Detecting and monitoring arsenic exposure accurately remains challenging, necessitating the development of sensitive detection methods. In this study, we introduce a novel approach using fast-scan cyclic voltammetry (FSCV) coupled with carbon-fiber microelectrodes (CFMs) for the electrochemical detection of As^3+^. Through an in-depth pH study using tris buffer, we optimized the electrochemical parameters for both acidic and basic media. Our sensor demonstrated high selectivity, distinguishing the As^3+^ signal from those of As^5+^ and other potential interferents under ambient conditions. We achieved a limit of detection (LOD) of 0.5 μM (37.46 ppb) and a sensitivity of 2.292 nA/μM for bare CFMs. Microscopic data confirmed the sensor’s stability at lower, physiologically relevant concentrations. Additionally, using our previously reported double-bore CFMs, we simultaneously detected As^3+^-Cu^2+^ and As^3+^-Cd^2+^ in tris buffer, enhancing the LOD of As^3+^ to 0.2 μM (14.98 ppb). To our knowledge, this is the first study to use CFMs for the rapid and selective detection of As^3+^ via FSCV. Our sensor’s ability to distinguish As^3+^ from As^5+^ in a physiologically relevant pH environment showcases its potential for future in vivo studies.

## 1. Introduction

Arsenic, a naturally occurring metalloid known as the “king of poisons”, has a well-documented history of toxicity, posing significant risks to human health due to its widespread presence in the environment and its use as a potent poison [1]. It exists primarily in trivalent (As^3+^ or arsenite) and pentavalent (As^5+^ or arsenate) forms, with the trivalent form being particularly toxic due to its high water solubility and slow excretion rate [2,3]. Anthropogenic sources of arsenic contamination include its use in insecticides, herbicides, medicines, electronics, and industrial manufacturing [4,5]. Additionally, being one of the most abundant elements in the Earth’s crust, arsenic is widely distributed in water, soil, and the atmosphere [6]. Chronic exposure to arsenic, mainly through contaminated drinking water and food supplies, leads to various serious health issues, including cardiovascular diseases, neurological disorders, and several types of cancer, such as skin, lung, bladder, and kidney cancer [7,8]. To address these life-threatening health hazards, there is an urgent need for rapid, sensitive, and cost-effective arsenic detection methods particularly suitable for in vivo monitoring.

Traditional methods for arsenic detection include atomic absorption spectroscopy [9], inductively coupled plasma–mass spectrometry [10], colorimetric assays [11], X-ray fluorescence spectroscopy [12], and Raman spectroscopy [13]. While these approaches offer high sensitivity and excellent limits of detection (LODs), they are limited to in vitro analysis, hindering real-time in vivo measurements. Moreover, they often require expensive equipment and time-consuming sample pre-treatment protocols that may alter chemical speciation, a vital component in determining metal toxicity [14,15]. Especially for arsenic, it is crucial to develop a method that can accurately and selectively distinguish As^3+^ from As^5+^. In contrast with those non-electrochemical methods, electrochemical tools offer a simple, rapid, and highly sensitive means of analysis while enabling the specification of metal speciation. Electrochemical sensors analyze electrical properties, such as potential, current, and conductivity resulting from a chemical reaction, to identify the analyte composition of a sample. A complete circuit consists of a working electrode, a reference electrode, and a counter electrode. While various types of electrochemical sensors exist, including potentiometric, amperometric, and conductometric sensors, voltammetric sensors are the most common due to their high selectivity, excellent sensitivity, versatility, faster response time, and ease of use. The fabrication process of electrochemical sensors varies depending on the type of sensor. Generally, a sensing material is deposited on a conductive substrate using various surface modification protocols to enhance the sensor’s sensitivity.

Anodic stripping voltammetry is commonly used in electrochemical sensors for arsenic detection, employing various electrodes such as platinum-disc [16], pencil graphite [17], silver [18], glassy carbon [19], and carbon nanotubes [20]. Additionally, differential pulse voltammetry [21] and cyclic voltammetry [22] have also been explored for arsenic detection. Given the pH-dependent nature of arsenic as seen below, it is crucial to carefully consider both the solution pH and the potential electrochemical oxidation–reduction reaction mechanism when conducting electrochemical experiments involving As^3+^/As^5+^ systems.

(1) $$AsO_{2(aq)}^{-}+2H_{2}O_{\left(l\right)}+3e^{-}⇌{As}_{\left(s\right)}+4OH_{\left(aq\right)}^{-}E{}^{\circ}=-0.68\;V$$

(2) $$AsO_{4(aq)}^{3-}+2H_{2}O_{\left(l\right)}+2e^{-}⇌AsO_{2(aq)}^{-}+4OH_{\left(aq\right)}^{-}E{}^{\circ}=-0.67\;V$$

(3) $$HAsO_{2(aq)}+3H_{\left(aq\right)}^{+}+3e^{-}⇌{As}_{\left(s\right)}+2H_{2}O_{\left(l\right)}E{}^{\circ}=0.24\;V$$

(4) $$H_{3}AsO_{4(aq)}+2H_{\left(aq\right)}^{+}+2e^{-}⇌HAsO_{2(aq)}+2H_{2}O_{\left(l\right)}E{}^{\circ}=0.56\;V$$

Interestingly, most of the reported electrochemical studies have been conducted under extreme pH conditions [18,20,22], making them unsuitable for measurements in physiological pH environments. Additionally, despite the reported ultra-low limits of detection achieved in vitro with existing electrochemical approaches, the temporal resolution of these studies is insufficient for real-time monitoring in living systems, thereby limiting the translatability of these sensors for in vivo studies.

To overcome these limitations, fast-scan cyclic voltammetry (FSCV) emerges as a promising electrochemical technique capable of providing rapid, real-time measurements of neurotransmitters and toxic heavy metals with a temporal resolution of 100 ms. By coupling FSCV with small, biocompatible carbon-fiber microelectrodes (CFMs), a potent electrochemical sensor can be fabricated, which is ideal for in vivo metal detection. CFMs offer distinct advantages due to the presence of surface oxide functional groups that readily adsorb many analyte ions, thereby enhancing sensitivity and selectivity in the detection process. FSCV-based metal sensors, when paired with CFMs, have demonstrated successful detection of Cu^2+^ [23], Pb^2+^ [24], and Cd^2+^ in tris buffer, simulating artificial cerebellum fluid (ACF) and artificial urine at physiologically relevant pH levels. These studies showcase the capability of FSCV-based metal sensors to function in environments mimicking biological fluids, facilitating the monitoring of metal concentrations in vivo under conditions closely resembling physiological settings.

In this study, we conducted a comprehensive investigation using CFMs and FSCV to detect As^3+^. Considering the pH-dependent aqueous chemistry of arsenic, we optimized the electrochemical parameters required to detect As^3+^ in both acidic and basic tris solutions. Selectivity tests demonstrated the excellent specificity of our approach in generating As^3+^-specific signals in the presence of As^5+^ and other interfering metal ions. We constructed a calibration curve to determine the LOD, sensitivity, and linear range of our sensor. Furthermore, we evaluated the stability of our sensor in the presence of low and high concentrations of arsenic, both electrochemically and microscopically. Additionally, we demonstrated that our optimized conditions enabled the co-detection of As^3+^ together with Cu^2+^ and Cd^2+^ using our previously reported double-bore CFMs [25], resulting in enhanced LOD and sensitivity compared with single CFMs. The ability of our sensor to detect As^3+^ under ambient conditions in tris buffer, which mimics ACF at a more physiologically relevant pH, with greater selectivity at a temporal resolution of 100 ms highlights its potential for future in vivo studies. Furthermore, to the best of our knowledge, this is the fastest electrochemical detection method reported to date for detecting As^3+^, facilitating easier real-time, in vivo monitoring in the future.

## 2. Materials and Methods

### 2.1. Reagents

Unless otherwise specified, all chemicals were purchased from Sigma-Aldrich (St. Louis, MO, USA). Sodium meta-arsenite (Alfa Aesar, MA, USA) was used as the As^3+^ source. As^3+^ solutions were prepared in tris buffer composed of tris hydrochloride (15 mM), NaCl (140 mM), KCl (3.25 mM), CaCl_2_ (1.2 mM), NaH_2_PO_4_ (1.25 mM), MgCl_2_ (1.2 mM), and Na_2_SO_4_ (2.0 mM) at varying pH levels (2.5–8.5). Cr(NO_3_)_3_·9H_2_O (Alfa Aesar, MA, USA), Fe(NO_3_)_3_·9H_2_O, Al(NO_3_)_3_·9H_2_O, and As_2_O_5_ were used as the sources for the selectivity test in tris buffer. Cadmium chloride (Alfa Aesar, MA, USA) and cupric sulfate were used as Cd^2+^ and Cu^2+^ sources for the double-bore CFM experiments.

### 2.2. Fabrication of Single-Bore CFMs

CFMs were constructed by inserting a single carbon fiber (diameter: 7 µm, GoodFellow, Pittsburgh, PA, USA) into borosilicate glass capillaries (internal diameter: 0.58 mm, external diameter: 1.0 mm, Sutter Instruments, Novato, CA, USA) using electrostatic forces between a wire and the carbon fibers. The fiber-filled capillaries were then pulled under gravity using a vertical micropipette puller, PE-100 (Narishige Group, Setagaya-Ku, Tokyo, Japan), resulting in a carbon–glass seal. Finally, the pulled CFMs were manually trimmed to 130–140 µm under an optical microscope.

### 2.3. Fabrication of Double-Bore CFMs

Following the method described by the Pathirathna group [25], two individual carbon fibers (diameter: 7 μm, Goodfellow, Pittsburgh, PA, USA) were inserted into two bores diagonally arranged in a four-bore borosilicate glass capillary (bore diameter of 0.015′′ and outer diameter of 0.062′′, Friedrich and Dimmock, Millville, NJ, USA). The fibers were held in place by electrostatic forces between a wire and the carbon fibers. Subsequently, the fiber-filled capillaries were pulled under gravity using a vertical puller, PE-100 (Narishige Group, Setagaya-Ku, Tokyo, Japan), resulting in two separate carbon–glass seals. Finally, the pulled CFMs were manually trimmed to 40–50 μm under an optical microscope (Unitron Examet-5 series, Commack, NY, USA).

### 2.4. Gold Nanoparticle Electrodeposition

Following the electrodeposition method described by the Zestos group [26], the surfaces of the CFMs were modified by electrodepositing gold nanoparticles. This was achieved by immersing CFMs in a solution containing 0.5 mM HAuCl_4_ mixed in 0.1 M KCl and cycling the potential from +0.2 V to −1.0 V at 50 mV/s for 10 cycles. The electrochemical setup consisted of a three-electrode system, with an in-house-built Ag/AgCl electrode serving as the reference electrode and a Pt wire (Alfa Aesar, MA, USA) as the counter electrode. The electrodeposition process was conducted using a CHI660E potentiostat (CH Instruments, Austin, TX, USA).

### 2.5. FSCV Electrochemical Measurements

All FSCV electrochemical measurements were conducted using a two-electrode system, employing CFMs as working electrodes and an in-house-built Ag/AgCl electrode as the reference electrode. Data collection, analysis, and background subtraction were performed using the Quad-UEI system (Electronics Design Facility, University of North Carolina, Chapel Hill, NC, USA).

### 2.6. Imaging with Scanning Electron Microscopy

CFMs were imaged using a scanning electron microscope (SEM) (JSM-6380/LV, Jeol Ltd., Tokyo, Japan) located at the High-Resolution Microscopy and Advanced Imaging Center at the Florida Institute of Technology. Images were captured at a magnification of 6500x, with electron beam energies set at 10 and 12 kV.

## 3. Results and Discussion

### 3.1. Optimization of Electrochemical Parameters in Acidic and Basic Media

Previously reported studies on arsenic detection using cyclic voltammetry mostly utilized relatively large electrodes and slow scan rates [21], resulting in a response primarily driven by diffusion. In contrast, CFMs are microelectrodes that operate at ultra-fast scan rates, making the fundamental mechanism behind our sensor adsorption-driven, as previously explained. To minimize potential interferences from a complex matrix, we initiated our experiments in a simple KCl solution. After optimizing waveform parameters, we were able to generate a distinct As^3+^-specific signal (Figure S1) using a bare CFM in KCl. Since our ultimate goal was to develop an electrochemical sensor capable of detecting As^3+^ in the brain, along with other toxic metal ions and neurotransmitters, we applied the same waveform in tris buffer. Tris buffer is commonly employed by electrochemists, especially in neurotransmitter studies, due to its resemblance to ACF. Therefore, conducting in vitro experiments in tris buffer is pertinent for future in vivo studies. Additionally, researchers, including ourselves, have successfully optimized FSCV parameters to detect Cu^2+^ [23], and Cd^2+^ in tris buffer using CFMs. Despite conducting an in-depth optimization study with bare CFMs in tris buffer, where we varied positive, negative, and resting potentials along with scan rates to generate a unique As^3+^-specific cyclic voltammogram (CV), we were unable to obtain such a signal. Subsequently, we modified our CFMs using previously reported surface modification strategies, specifically electrodepositing polydopamine [27] and gold nanoparticles [26], followed by optimization of electrochemical parameters to observe if we could obtain a unique As^3+^ CV. However, we did not observe any promising, reproducible CVs.

**Figure 1:**
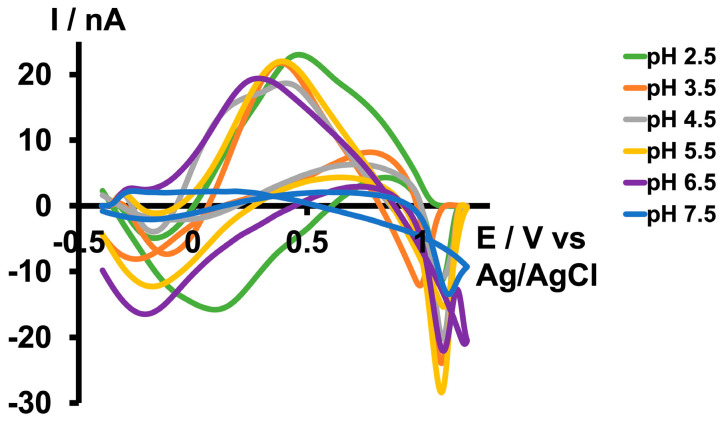
Representative CVs obtained for 10 µM As^3+^ in tris buffer at pH 2.5 (green), 3.5 (orange), 4.5 (grey), 5.5 (yellow), 6.5 (purple), and 7.5 (blue).

In addition to the differences in matrix complexity between tris buffer and KCl, another significant distinction was the pH of these two solutions. While the pH of tris buffer was adjusted to 7.4 to mimic ACF, the pH of the KCl solution used to generate a unique As^3+^-specific CV was approximately 5.0. Considering the significant impact of aqueous chemistry on the As^3+^/As^5+^ equilibrium based on the pH of the medium (as demonstrated in Equations (1)–(4)), we conducted a comprehensive pH study in tris buffer. Initially, we lowered the pH of the tris buffer to 2.5 by adding HCl and optimized the electrochemical parameters until we obtained a unique CV for As^3+^ in the tris buffer (Figure 1). On the forward scan, an oxidation peak was observed at ~0.4 V followed by a reduction peak at ~0.1 V on the backward scan when scanning from −0.4 V to 1.2 V with a resting potential of −0.4 V at a scan rate of 400 V/s. The switching peak observed at the positive potential terminal was attributed to the change in capacitance of the double layer at faster scan rates [28].

**Figure 2:**
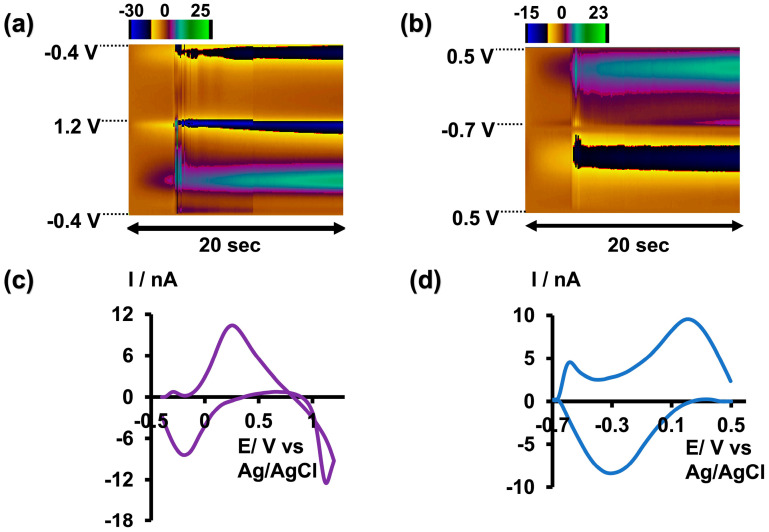
(**a**) Representative color plot obtained for 5 µM As^3+^ in tris buffer at pH 6.5. (**b**) Representative color plot obtained for 10 µM As^3+^ in tris buffer at pH 8.5. (**c**) Representative CV obtained for 5 µM As^3+^ in tris buffer at pH 6.5. (**d**) Representative CV obtained for 10 µM As^3+^ in tris buffer at pH 8.5.

Subsequently, we increased the pH of the tris buffer by adding NaOH. As depicted in Figure 1, we were able to replicate the same CV until a pH of 6.5 with minimal potential shift in the forward oxidation peak. However, the CV disappeared at pH 7.5. Since we did not obtain a distinct CV with the same waveform we used at pH 2.5 above 7.5, we then attempted to optimize another waveform under basic conditions. The maximum pH we could achieve in the tris buffer was 8.5, as some of the matrix constituents began to precipitate above this pH. Interestingly, as illustrated in Figure 2b,d, instead of observing an oxidation peak during the forward scan, we observed a reduction peak at around −0.3 V and an oxidation peak during the backward scan at approximately 0.2 V. This occurred while scanning from 0.5 V to −0.7 V with a resting potential of 0.5 V at a scan rate of 400 V/s. The difference between the two CVs obtained under acidic and basic conditions may be attributed to the variations in aqueous chemistry at these two pH levels. 

Given that arsenic poisoning can often lead to severe gastrointestinal symptoms and increased acidity of blood and body tissues (acidosis) [29], using tris buffer with a pH of 6.5 would maintain physiological relevance without compromising oxidation current readings. Therefore, future studies employing this slightly acidic As^3+^-specific waveform will be conducted in tris buffer at a pH of 6.5 (Figure 2a,c). 

**Figure 3:**
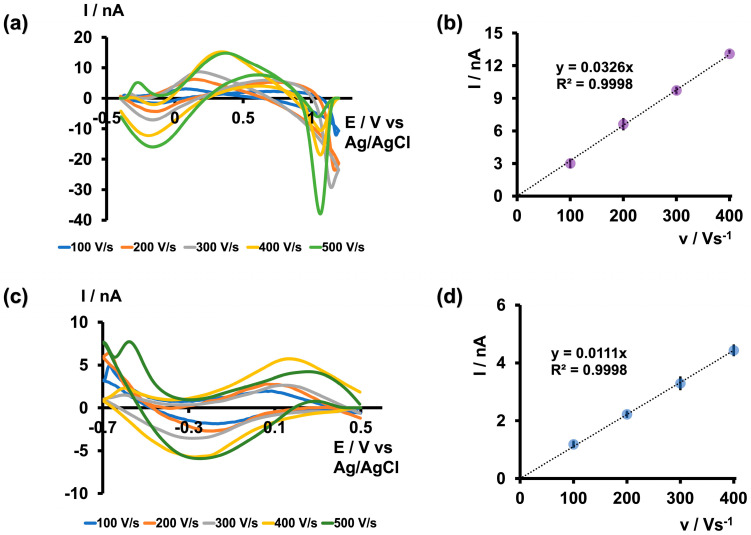
(**a**) Representative CVs obtained for 5 µM As^3+^ at each scan rate when the potential was varied from −0.4 V to 1.2 V and back to −0.4 V and (**b**) plot of maximum oxidation peak current vs. scan rate in tris buffer at pH 6.5. (**c**) Representative CVs obtained for 5 µM As^3+^ for each scan rate when the potential was varied from 0.5 V to −0.7 V and back to 0.5 V and (**d**) plot of maximum reduction peak current vs. scan rate in tris buffer at pH 8.5. Each data point represents the average oxidation current ± standard error of the mean obtained for three CFMs with at least 4 replicate measurements for each CFM (minimum of 12 total replicates).

After determining the maximum pH in both acidic and basic media that could produce unique As^3+^-specific CVs in tris buffer, along with optimizing the positive, negative, and resting potentials, we further varied the scan rate from 100 to 500 V/s to identify the optimal scan rate. As depicted in Figure 3 and Figure S2, the current readings increased up to 400 V/s in both acidic and basic media within their respective potential windows, plateauing at 500 V/s. However, the CVs obtained at 500 V/s appeared broader and distorted compared with those at 400 V/s, leading us to select 400 V/s as the optimal scan rate. Additionally, the R^2^ values of the two plots depicting the scan rate versus current were approximately 0.99, indicating an adsorption-driven response in both acidic and basic media. Moreover, this relationship between scan rate and current closely resembles what has been observed in previously reported FSCV metal sensors [23,24].

### 3.2. Selectivity Test

**Figure 4:**
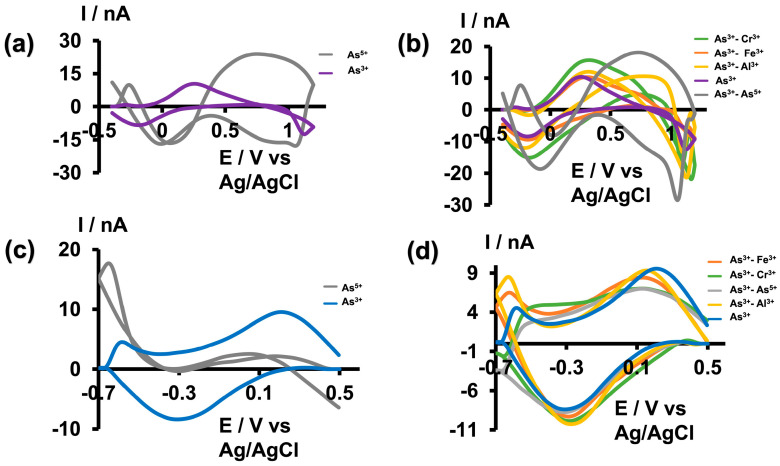
(**a**) Representative CVs obtained for 500 µM As^5+^ (gray) and 5 µM As^3+^ (purple) in tris buffer at pH 6.5. (**b**) Representative CVs obtained for 5 µM As^3+^ in the presence of 500 µM Cr^3+^ (green), Fe^3+^ (orange), Al^3+^ (yellow), As^5+^ (grey), and As^3+^ alone (purple) in tris buffer pH 6.5. (**c**) Representative CVs obtained for 500 µM As^5+^ (gray) and 5 µM As^3+^ (blue) in tris buffer at pH 8.5. (**d**) Representative CVs obtained for 5 µM As^3+^ in the presence of 500 µM Fe^3+^ (orange), Cr^3+^ (green), As^5+^ (grey), Al^3+^ (yellow), and As^3+^ only (blue) in tris buffer with pH 8.5.

As metal toxicity varies depending on the speciation of the metal, it is crucial to evaluate a metal sensor’s ability to selectively detect not only one specific metal ion among other interfering metal ions, but also among different species of the same metal ions. It has been found that As^3+^ is approximately 5–10 times more toxic than As^5+^ due to its high water solubility and low excretion rates within the body [3,7]. Therefore, quantitative and qualitative detection of As^3+^ is vital for assessing the toxicity of ingested arsenic within the body. While many previously reported electrochemical studies claim that oxidation/reduction peaks originate from the presence of arsenic, a comparison between As^3+^ and As^5+^ studies is often lacking [16,17,18,19,21]. Furthermore, some studies were conducted in the presence of nitrogen to prevent the possible oxidation of As^3+^ to As^5+^ in the presence of oxygen [17,30,31]. However, nitrogen purging is not feasible for in vivo, real-time monitoring. Therefore, we did not use any special precautions to prevent this possible oxidation when optimizing detection parameters. To further confirm that the observed CVs were solely due to the presence of As^3+^, we tested our sensor against As^5+^, Cr^3+^, Fe^3+^, and Al^3+^. Initially, we tested As^5+^ (500 µM) prepared in tris buffer at pH 6.5 and 8.5 using the optimized waveforms. As depicted in Figure 4a,c, no distinct CVs were obtained; instead, distorted and indistinct signals appeared. Subsequently, we prepared solution mixtures by mixing As^3+^ with other interfering ions while maintaining a concentration ratio of 1:100 for As^3+^ to other interfering ions (5:500 µM) in tris buffer at pH 6.5 and 8.5. As illustrated in Figure 4b,d, all solution mixtures resulted in As^3+^ CVs with slight shifts and negligible distortions. This demonstrates the greater selectivity of our sensor towards As^3+^ under both solution conditions using the unique waveforms. Furthermore, as our tris buffer already contains high concentrations of Ca^2+^, Mg^2+^, and Na^+^, we did not perform additional selectivity tests with these commonly found ions.

### 3.3. Calibration Study

**Figure 5:**
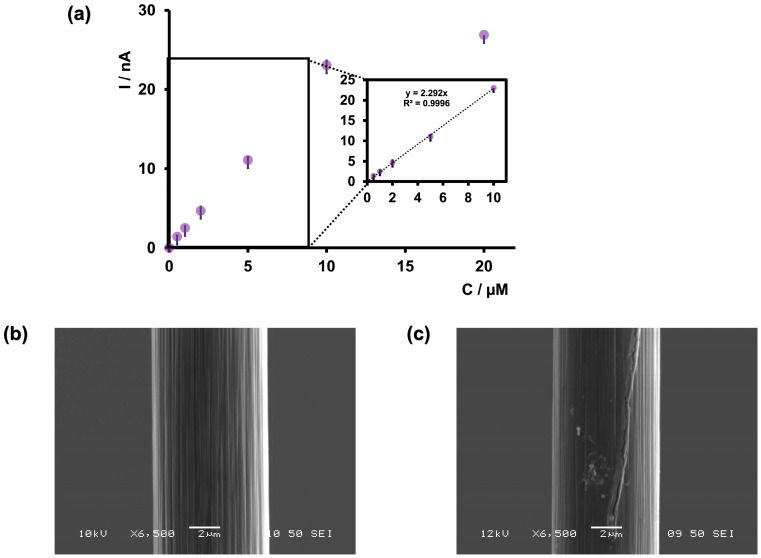
(**a**) Calibration curve of As^3+^ using bare CFMs and FSCV. The potential was cycled from −0.4 to +1.2 V at 400 V/s in tris buffer at pH 6.5. The inset of the graph represents the linear range of the calibration plot. (**b**) SEM image of bare CFM. (**c**) SEM image of CFM after collecting FSCV measurements of 5 µM As^3+^.

Anticipating that our future in vivo studies will be conducted under slightly acidic conditions, especially as arsenic ingestion results in acidosis, we conducted our calibration study only at pH 6.5 in tris buffer. As shown in Figure 5a, the maximum oxidation current increased up to 10 μM and then plateaued. The LOD was found to be 0.5 μM (37.46 ppb), with a sensitivity of 2.292 nA/μM. Excitingly, as shown in Table 1, the LOD of our sensor was comparable to that of previously reported electrochemical sensors for arsenic detection. Moreover, this LOD was achieved in a physiologically relevant buffer at a pH of 6.5, making our sensor ideal for future development as an arsenic detection tool, particularly for measurements in the brain.

We also observed that CFMs tended to foul at higher concentrations (above 10 μM). Therefore, we examined the surface of our CFMs before and after an FSCV experiment via SEM to analyze any visible changes in surface morphology. As depicted in Figure 4b, a clear surface was visible before an experiment, whereas arsenic deposition could be seen after an experiment (Figure 4c), confirming our electrochemical observations. Furthermore, we tested the stability of our sensor at 1 μM over 20 consecutive injections, and the sensor demonstrated excellent stability (Figure S3).

Additionally, because our LOD was not as low as that of some other reported sensors and gold has been incorporated into those sensors to improve the sensitivity of arsenic detection [20,32,35], we attempted to modify our bare CFMs by electrodeposition of gold nanoparticles [26] (Figure S4) using one of our previously successful surface modification strategies for Cd^2+^ detection. Interestingly, upon modifying the electrode surface with gold nanoparticles, the sensor response decreased. Although this surface modification on CFMs has been successful in the past [26], this method might not be effective under acidic conditions at faster scan rates to detect As^3+^. 

### 3.4. Co-Detection of As^3+^ with Toxic Heavy Metals

**Figure 6:**
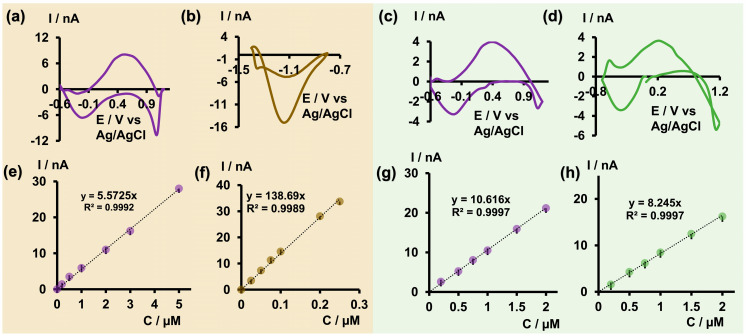
(**a**) Representative CV obtained for 2 μM As^3+^ (**b**) and 0.1 μM Cd^2+^ in As^3+^-Cd^2+^ solution mixture, and (**c**) representative CV obtained for 0.5 μM As^3+^ (**d**) and 0.5 μM Cu^2+^ in As^3+^-Cu^2+^ solution mixture in tris at pH 6.5 when co-detecting using double-bore CFMs. Corresponding calibration curves obtained in As^3+^-Cd^2+^ solution mixtures for As^3+^ and Cd^2+^ are depicted in (**e**,**f**) while those obtained for As^3+^ and Cu^2+^ in As^3+^-Cu^2+^ solution mixture are represented in (**g**,**h**).

After optimizing the electrochemical parameters and establishing the analytical parameters to detect As^3+^ with bare CFMs, we decided to apply the optimized waveform to test the feasibility of co-detecting As^3+^ with other toxic metal ions, such as Cu^2+^ and Cd^2+^, at ultra-fast temporal resolution. In a previous study, we pioneered the fabrication of a double-bore CFM capable of simultaneously detecting neurotransmitters and Cu^2+^ [25] using FSCV, demonstrating enhanced sensitivity compared with a single CFM. For this study, we performed our experiments in tris buffer at pH 6.5 facilitating the detection of arsenic. Additionally, we selectively modified one electrode in our double-bore assembly by electrodepositing it with gold nanoparticles, necessary for Cd^2+^ detection. Before conducting FSCV measurements, we tested whether our double-bore CFMs could still maintain a stable nanogap with these new modifications (Figure S5). Once the gap was confirmed, we performed FSCV measurements in solution mixtures of As^3+^-Cd^2+^ and As^3+^-Cu^2+^ by varying the concentrations of each metal ion and constructed calibration curves (Figure 6). During co-detection, a slight distortion of the original CVs was anticipated [25]. Interestingly, as seen in Figure 6a,c, the characteristic shape and oxidation of As^3+^ were observed for both solution mixtures. Similarly, Cd^2+^ and Cu^2+^ maintained their unique shapes [23] and characteristic reduction peaks with minimal distortion (Figure 6b,d).

Moreover, as reported previously with double-bore CFMs [25], improved sensitivity and LODs were achieved for As^3+^ and Cu^2+^ upon co-detection. The LOD of As^3+^ was improved to 0.2 μM (14.98 ppb) in both analyte mixtures. Similarly, the LOD of Cu^2+^ was improved from the previously reported 0.5 μM [23] with single-bore CFMs to 0.2 μM, despite the change in buffer pH. Conversely, the LOD of Cd^2+^ decreased to 0.025 μM from the 0.01 μM reported for single-bore CFMs. We attribute the enhanced sensitivity to the presence of a secondary electric field in close proximity, which can capture and cycle the products of one oxidation/reduction reaction back to the reactants, thus preventing diffusion away from the electrode’s surface [25]. The decreased LOD in Cd^2+^ may be due to changes in the pH of the tris buffer, altering the free Cd^2+^ presence in the medium, as well as changes in surface modification with gold nanoparticles. The ability to co-detect As^3+^ together with other metal ions at a higher temporal resolution is not only important for future in vivo studies, but will also greatly benefit the development of environmental monitoring capable of co-detecting toxic metal ions with excellent selectivity and sensitivity.

## 4. Conclusions

The extreme toxicity of arsenic, combined with its increasing abundance, underscores the urgent need for the development of a sensor capable of ultra-fast and selective detection of ultra-low arsenic concentrations. Traditional methods of arsenic detection rely on laborious and time-consuming processes, often requiring sophisticated equipment and extensive sample preparation. Moreover, these methods may alter the chemical speciation of arsenic, leading to inaccurate results. Additionally, electrochemical techniques commonly used for arsenic detection suffer from limitations such as a lack of selectivity for the more toxic As^3+^ species and poor translatability to in vivo applications due to the requirement of extreme pH conditions. Furthermore, all these methods lack the required temporal resolution for successful in vivo measurements, particularly in the brain.

In this study, we conducted a comprehensive investigation using CFMs to detect As^3+^ at an ultra-fast temporal resolution using FSCV. We performed an in-depth pH study to better understand the complex aqueous chemistry of arsenic, allowing us to optimize the electrochemical parameters needed for both acidic and basic media. Subsequently, we evaluated the selectivity of our sensor by conducting a series of FSCV readings for As^3+^ in the presence of potential interfering ions, including As^5+^. After identifying the optimal pH and waveform combination, we constructed a calibration curve to determine analytical parameters, including linear range and LOD. Stability tests and SEM images confirmed that our sensor remained stable at lower physiologically relevant concentrations of As^3+^, while the sensor became unstable at higher concentrations due to increased fouling. Furthermore, we applied our previously reported double-bore CFMs to the co-detection of As^3+^ and toxic heavy metals using these optimal conditions.

Excitingly, the co-detection of As^3+^ with Cd^2+^ and Cu^2+^ was successful, despite the change in buffer pH required for arsenic detection. Additionally, our double-bore CFMs remained intact upon the electrodeposition of gold nanoparticles on one carbon fiber for the detection of Cd^2+^, showcasing great potential for future multi-bore studies wherein each carbon fiber will be surface-modified for each specific analyte. Furthermore, the LOD obtained for both single and double-bore sensors retains physiological relevance to the confirmed exposure limit for arsenic.

This study demonstrates several strengths, including the comprehensive optimization of arsenic detection at physiologically relevant pH levels, operating at ultra-fast scan rates with high temporal resolution. Additionally, this study successfully detected the more toxic As^3+^ species over As^5+^ and other potential interfering ions. Calibration studies maintain physiological relevance for future in vivo studies. However, a relatively low stability of our sensor compared with other FSCV-based metal sensors reported is a weakness. Similarly, a critical challenge is the relatively high LOD, necessitating further exploration of surface modification strategies for improved performance. Opportunities for this electrochemical sensor include the potential development of multi-bore CFMs and alternative surface modification techniques to expand the sensor’s capabilities. Additionally, some threats include competition from existing or emerging sensor technologies and regulatory hurdles for in vivo application. To the best of our knowledge, this is the first study to report the use of both single and double-bore CFMs for the electrochemical detection of As^3+^ via FSCV under ambient conditions and complex matrices, showcasing the power of our sensor to be fabricated as a future in vivo sensor. 

## Figures and Tables

**Table 1:** Comparison of previously reported electrochemical arsenic sensors.

Electrochemical Method	Sensor	LOD (ppb)	Sensitivity	Matrix/Buffer	Reference
Square-Wave Anodic Stripping Voltammetry	Gold nanoparticle decorated nanorod	0.019	16.268±0.242 μA ppb^−1^ cm^−2^	0.1 M Na_2_CO_3_-NaHCO_3_ (pH 9)	[30,32]
Square-Wave Anodic Stripping Voltammetry	Magnetite decorated gold nanoparticles modified glassy carbon electrode	0.22	0.122 mA ppb^−1^	0.2 M Acetate Buffer (pH 5)	[30]
Anodic Stripping Voltammetry	Nanogold modified glassy carbon electrode	0.28	Not reported	0.1 M H_2_SO_4_	[19]
Cyclic Voltammetry	Iridium-implanted boron-doped diamond electrodes	1.5	93 nA μM^−1^ cm^−2^	0.1 M Phosphate Buffer Solution (pH 4)	[33]
Cyclic Voltammetry	Glassy carbon electrode modified with cobalt oxide nanoparticles	8.24	111.3 nA μM^−1^	0.1 M Phosphate Buffer Solution (pH 7)	[34]
Differential Pulse Voltammetry	Goethite nanoparticles wrapped on reduced graphene oxide nanosheet	22.84	0.39 μ$A^{-1}\;\mu$gL^−1^	0.1 M Phosphate Buffer Solution (pH 5)	[21]
Square-Wave Voltammetry	Glassy carbon electrode modified with gold nanoparticles on multiwalled carbon nanotubes	32.63	1985 μA μM^−1^	0.1 M HCl	[20]
Fast-Scan Cyclic Voltammetry	Carbon fiber microelectrodes	37.46	2.292 nA μM^−1^	Tris Buffer (pH 6.5)	
Anodic Stripping Voltammetry	Silver electrode	47.2	2.6 A M^−1^	0.1 M HNO_3_	[18]
Anodic Stripping Voltammetry	Gold nanoparticle array	59.93	0.91 C M^−1^	1 M H_2_SO_4_	[35]
Cyclic Voltammetry	Iridium-modified boron-doped diamond electrode	347.63	0.056 μA μM^−1^ cm^−2^	0.1 M Phosphate Buffer Solution (pH 3)	[36]

## Data Availability

All the necessary data are available in the article.

## Supplementary Materials

The following supporting information can be downloaded at: https://www.mdpi.com/article/10.3390/mi15060733/s1, Figure S1: Detection of As^3+^ in 0.1 M KCl; Figure S2: Optimization of scan rate under acidic and basic conditions; Figure S3: Stability test under acidic conditions; Figure S4: Comparison of As^3+^ response on bare CFM and gold nanoparticle-modified CFM; Figure S5: Oscilloscope images of the double-bore CFMs in As^3+^-Cd^2+^ and As^3+^-Cu^2+^ solutions.
