# Supplementary material for: Ultrafast Detection of Arsenic Using Carbon-Fiber Microelectrodes and Fast-Scan Cyclic Voltammetry
Source: Micromachines (Basel). 2024 May 31;15(6):733. doi: 10.3390/mi15060733 (PMC11205817; doi:10.3390/mi15060733)
Supplement: Supplementary file 1 [file micromachines-15-00733-s001.zip › micromachines-3013032-supplementary.pdf]

Supplementary Material:

Ultrafast Detection of Arsenic using Carbon-Fiber Microelectrodes and Fast-Scan Cyclic Voltammetry

Noel Manring[1], Miriam Strini[1], Jonathan Xavier, Jessica L. Smeltz[1], Pavithra Pathirathna[1*]

[1]Department of Chemistry and Chemical Engineering, Florida Institute of Technology, 150 W. University Blvd, Melbourne, FL, 32901, USA.

***Correspondence: ppathirathna@fit.edu**

Table of Contents:

1. Detection of As^{3+} in 0.1 M KCl
2. Optimization of scan rate in acidic and basic conditions
3. Stability test in acidic conditions
4. Comparison of As^{3+} response on bare CFM and gold nanoparticle-modified CFM
5. Oscilloscope images of the double-bore CFMs in As^{3+}-Cd^{2+} and As^{3+}-Cu^{2+} solutions

Detection of As^{3+} in 0.1 M KCl: We initiated our optimization studies in a simple KCl solution as this is the first time fast-scan cyclic voltammetry (FSCV) has been used with carbon-fiber microelectrodes (CFMs) to detect As^{3+} at ultra-fast temporal resolution. As illustrated in Figure S1, we observed an identifiable oxidation peak on the forward scan and a reduction peak on the backward scan for 10 μM As^{3+} when scanning from -0.6 V to 1.3 V with a resting potential of -0.6 V in 0.1 M KCl.

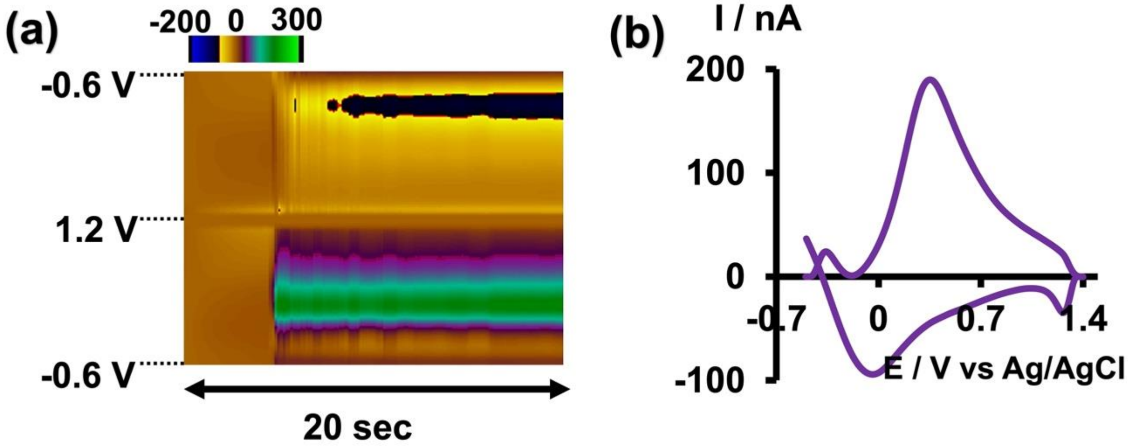

Figure S1. Representative (a) color plot and (b) CV for 10 μM As^{3+} in 0.1 M KCl.

Optimization of scan rate in acidic and basic conditions: After optimizing the positive, negative, and resting potentials, we explored various scan rates from 100 V/s to 500 V/s to identify the optimal scan rate for generating the maximum oxidation/reduction currents and a non-distorted As^{3+} CV for both acidic and basic tris solutions. As depicted in Figure S2, the oxidation current for As^{3+} in both conditions increased up to 400 V/s, plateauing at 500 V/s. Although the current at 500 V/s was similar to that at 400 V/s, the CV shape appeared distorted; therefore, 400 V/s was chosen as the optimal scan rate.

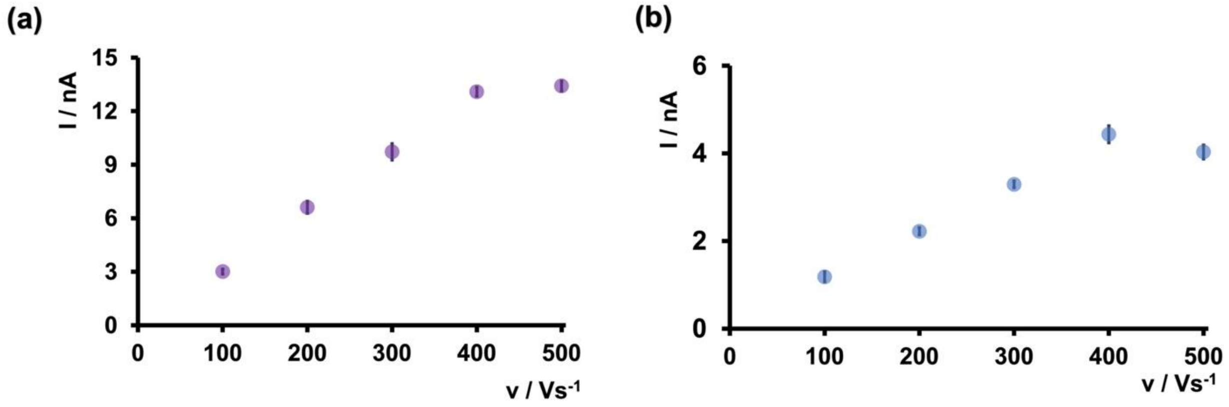

Figure S2. (**a**) The plot of maximum oxidation current vs. scan rate for 5 µM As^{3+} in tris buffer pH 6.5 upon cycling the potential was from -0.4 V to 1.2 V and back to -0.4 V. (**b**) The plot of maximum reduction current vs. scan rate for 5 µM As^{3+} in tris buffer pH 8.5 upon cycling the potential was from 0.5 V to -0.7 V and back to 0.5 V. Each data point represents the average reduction current ± standard error of mean obtained for three CFMs with at least four replicate measurements for each CFM (minimum of 12 total replicates).

Stability test in acidic conditions: We assessed the sensitivity of our sensor by consecutively injecting As^{3+}. As depicted in Figure S3, after 20 consecutive injections of 1 μM As^{3+}, the maximum oxidation current remained stable, indicating the excellent stability of our sensor.

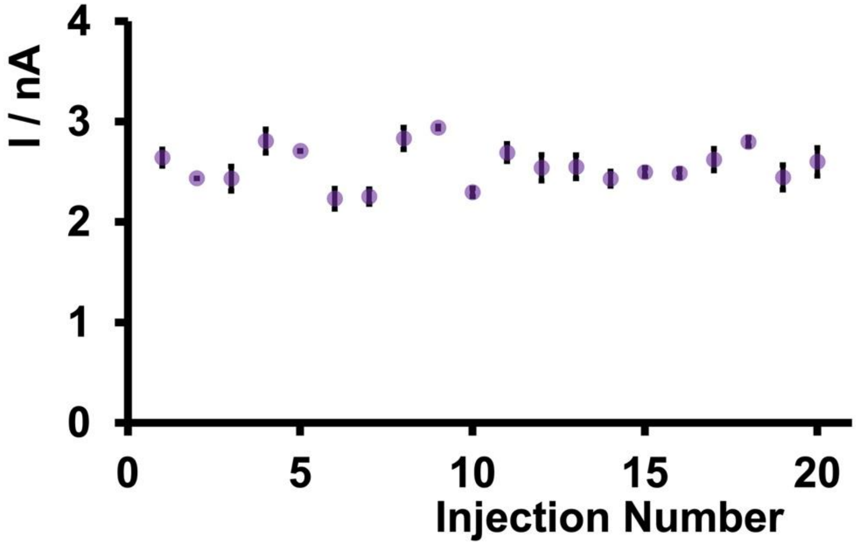

Figure S3. Maximum FSCV oxidation current obtained upon successive injections of 1 μM As^{3+} onto CFMs in tris buffer at pH 6.5. Each data point represents the average oxidation current ± standard error of the mean obtained for three CFMs.

Comparison of As^{3+} response on bare CFM and gold nanoparticle-modified CFM: To enhance the sensing capability of our sensor, we modified CFMs by electrodepositing gold nanoparticles. However, as shown in Figure S4, there was no increase in the oxidation current of As^{3+} CV; instead, the response decreased. Therefore, we did not perform further experiments with surface-modified CFMs.

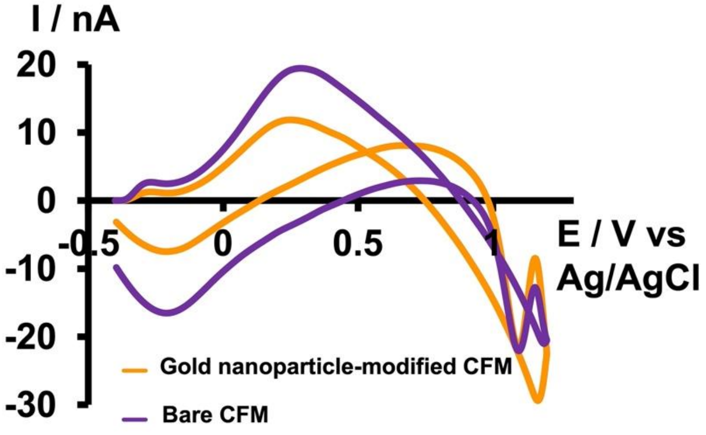

Figure S4. (**a**) Representative CVs obtained for 10 μM As^{3+} with gold nanoparticle-modified CFM (orange) and 10 μM As^{3+} with bare CFM (purple) using acidic waveform in tris buffer pH 6.5.

Oscilloscope images of the double-bore CFMs in As^{3+}-Cd^{2+} and As^{3+}-Cu^{2+} solutions: Before conducting co-detection of As^{3+} with Cd^{2+} and Cu^{2+} using double-bore CFMs, we confirmed the stability of the CFMs and tested whether they could maintain a stable gap between the two electrodes. Additionally, we modified one of the CFMs with gold nanoparticles to enable Cd^{2+} detection. As shown below, both double-bore CFM assemblies maintained the gap when different potential windows were applied.

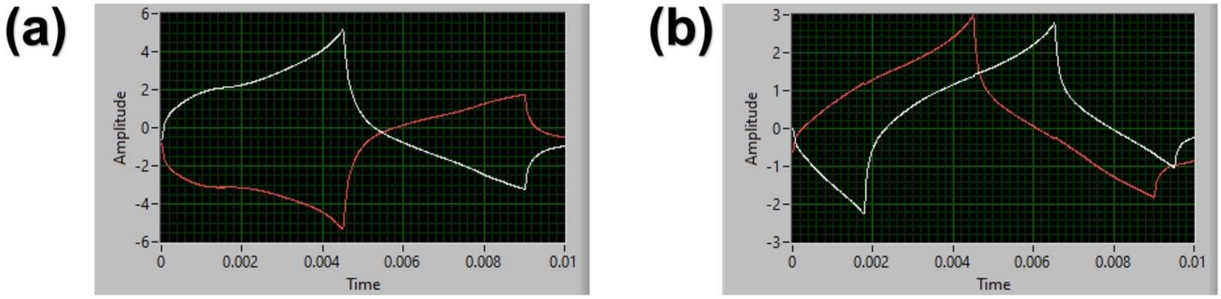

Figure S5. Oscilloscope images when two separate waveforms applied to double-bore CFMs in As^{3+}-Cd^{2+} and As^{3+}-Cu^{2+} solutions prepared in tris buffer at pH 6.5. (**a**) Red line corresponds to the Cd^{2+} waveform (−0.8 V to -1.4 V at 400 V/s) and the white line to the As^{3+} waveform (−0.4 V to +1.2 V at 400 V /s). (**b**) Red line corresponds to As^{3+} waveform (−0.4 V to +1.2 V at 400 V /s) and the white line corresponds to Cu^{2+} waveform (−0.7 V to +1.2 V at 400 V/s).
